# Improving Validity of Informed Consent for Biomedical Research in Zambia Using a Laboratory Exposure Intervention

**DOI:** 10.1371/journal.pone.0108305

**Published:** 2014-09-25

**Authors:** Joseph Mumba Zulu, Mpala Mwanza Lisulo, Ellen Besa, Patrick Kaonga, Caroline C. Chisenga, Mumba Chomba, Michelo Simuyandi, Rosemary Banda, Paul Kelly

**Affiliations:** 1 Departments of Community and Internal Medicine, University of Zambia (School of Medicine), Lusaka City, Lusaka Province, Zambia; 2 Department of Infectious and Tropical Diseases, London School of Hygiene and Tropical Medicine, London, United Kingdom; University of Washington, United States of America

## Abstract

**Background:**

Complex biomedical research can lead to disquiet in communities with limited exposure to scientific discussions, leading to rumours or to high drop-out rates. We set out to test an intervention designed to address apprehensions commonly encountered in a community where literacy is uncommon, and where complex biomedical research has been conducted for over a decade. We aimed to determine if it could improve the validity of consent.

**Methods:**

Data were collected using focus group discussions, key informant interviews and observations. We designed an intervention that exposed participants to a detailed demonstration of laboratory processes. Each group was interviewed twice in a day, before and after exposure to the intervention in order to assess changes in their views.

**Results:**

Factors that motivated people to participate in invasive biomedical research included a desire to stay healthy because of the screening during the recruitment process, regular advice from doctors, free medical services, and trust in the researchers. Inhibiting factors were limited knowledge about samples taken from their bodies during endoscopic procedures, the impact of endoscopy on the function of internal organs, and concerns about the use of biomedical samples. The belief that blood can be used for Satanic practices also created insecurities about drawing of blood samples. Further inhibiting factors included a fear of being labelled as HIV positive if known to consult heath workers repeatedly, and gender inequality. Concerns about the use and storage of blood and tissue samples were overcome by a laboratory exposure intervention.

**Conclusion:**

Selecting a group of members from target community and engaging them in a laboratory exposure intervention could be a useful tool for enhancing specific aspects of consent for biomedical research. Further work is needed to determine the extent to which improved understanding permeates beyond the immediate group participating in the intervention.

## Background

Successful recruitment and retention of subjects in clinical trials contributes to both the statistical power and the credibility of a trial [1]. However, this can be challenging in clinical trials which involve invasive procedures, particularly those outside the most familiar activities of medical care. Studies have shown that some participants in clinical research are reluctant to take part in such clinical trials because of misconceptions and fears regarding the use of blood samples [2].

These concerns and misconceptions, which are often grounded within a specific social-cultural context, generate rumours which negatively affect recruitment and increase losses to follow-up [3]. Loss to follow-up can happen even after consent is obtained [4] and may represent withdrawal of consent. For example, studies conducted in Ghana and Zambia confirmed the role culture plays in shaping perceptions and attitudes towards peoples’ involvement in studies that involve drawing of blood samples [5], [6]. Rumours about blood thefts by ‘Satanists’ contributed to high losses to follow-up in a trial conducted in Zambia on iron and multi-micronutrient supplementation. This was as a result of “belief of the existence of a cult which drinks human blood as part of their rituals, and such collection of blood was generally viewed with much suspicion” [6].

Discontent with the quantity of blood that people are requested to give may also cause people not to participate in clinical trials or withdraw from trials that involve drawing of blood samples [3]. Studies conducted in India showed that factors such as quality of facilities, convenience of the facilities where samples are drawn, and the quality of service provided in these facilities, also influenced people’s attitude towards blood donation [7]–[9]. Failure to adequately explore and understand the underlying contextual factors, for example social, cultural, economic and political issues, regarding drawing of blood and tissue samples could adversely affect programmes or laboratory investigations that require the use of the blood and tissue specimens [3], [10], [11].

This study aimed at understanding the community’s perceptions regarding involvement in complex biomedical research which requires collecting blood and intestinal biopsy specimens for advanced (non-routine) laboratory investigations in a community in the southern part of Lusaka, Zambia. Over 30 years, Zambia’s health systems have been stressed by the double burden of disease (communicable and non communicable), and in particular the health burdens of HIV infection which has a nationwide prevalence of about 13.5% [12]. Previous studies have suggested that biomedical research in Zambia, which often entails the participation of people with little or no education in science-related subjects, can lead to disquiet which leads to rumours or to high drop-out rates [6], [11]. In an attempt to enhance our participants’ understanding of, and engagement in, our research, we designed an intervention aimed at exposing participants to a detailed demonstration of laboratory processes over the course of a whole day. Here we report the results of an assessment of the impact of this intervention on their understanding of, and motivation to engage in, complex biomedical research in the community where we carry out such studies.

Two research questions were addressed. First, what factors motivate people to participate in a study which requires invasive procedures such as drawing of blood and taking biopsy specimens? Second, what factors might inhibit community members from participating in a study which requires undergoing such invasive procedures? We then assessed the impact of a laboratory exposure intervention.

## Methodology

### The study design

The study used a qualitative case study methodology to understand the community’s beliefs and perspectives about giving blood and biopsy specimens for research purposes. The case study methodology is an empirical approach that investigates contemporary phenomena within a real-life context; when the boundaries between phenomena and context are not clearly evident; and in which multiple sources of evidence are used [13]. The case study approach was considered appropriate for the study because research participants live within a complex context in a crowded underprivileged neighbourhood, which involves social interactions and relationships that subsequently influence the decisions regarding involvement of individuals in clinical trials. Participants in the current study were self-selected by volunteering during focus group discussions which had been held as part of the consent process for inclusion in a study of vaccines for diarrhoeal diseases (ISRCTN89702061). We did not include people who did not come forward for that study. In the previous/original study, the participants were recruited following a house-to-house sensitisation drive, then community discussions and face-to-face interviews. Recruitment processes for this study followed procedures described in previous publications [14], [15].

### Study Site

The study was conducted in Misisi compound, which is an unplanned shanty area close to the city centre with an estimated population of about 60,000 people. It is has a high housing density with poor shelter, sanitation, roads, water supply and limited health services. Like other compounds in Lusaka, the area is faced with several health related problems which include cholera, diarrhoea, malaria, cancer and HIV/AIDS. St. Lawrence health facility is located in Misisi compound. The facility is located in a church compound and is primarily orientated towards a large community malnutrition/HIV programme for children.

### Data collection methods

#### Focus Group Discussion

Four focus group discussions were conducted with residents of Misisi Compound, a residential area characterised by poor amenities, low socio-economic status, and low literacy levels. The discussions were conducted at the University Teaching Hospital, in the Department of Internal Medicine. Only four FGDs were held because we reached a point at which no new information was being obtained (data saturation). Two FGDs had four participants while the other two had only three participants. Having few people in the FGDs resulted in free and searching discussions. In total 14 people participated in the FGDs and the composition of the group is reflected in Table 1. Less than half (6) of the participants had previously been involved in a clinical trial. The FGD participants were aged from 20 years to 60 years. Their education was modest in that none of them had stayed in school beyond junior secondary school level (Grade 9). Each group was interviewed twice in one day.

**Table 1:** FGD Study participants.

FGD No.	Total No. ofparticipants	No. of femaleParticipants	No. of maleparticipants	Length of time of involvementin the study – average
1	4	3	1	4 years
2	4	3	1	3 years
3	3	2	1	1 year
4	3	0	3	1 year
Total	14	8	6	

The FGDs were conducted by an independent social scientist (JMZ) who had not been involved in the design or conduct of the vaccine study. The social scientist had postgraduate training and experience in qualitative research. No other people were present at the focus groups besides the facilitator and the participants. To ensure that no information was missed, the discussions were recorded digitally.

FGD was selected as the main method of data collection. It has proven to be a fast and efficient way of obtaining a wide variety of information in a relatively short period of time. It is also an effective way of gathering information on sensitive topics because they enhance the disclosure of more data or material in three ways: awareness of shared experience may encourage discussions on difficult and sensitive issues; agreement between group members can build an elaborated and fuller landscape of views; disagreement between group members may lead participants to defend their views and provide further explanation [16].

Two FGDs were conducted for each group. The first discussion was held before the laboratory exposure intervention while the second discussion was held after it. The first FGD focused on understanding personal and community views regarding: 1) the use to which biological specimens are put after collection, 2) issues relating to storage of specimens, 3) willingness to donate specimens among community members (including themselves), and 4) barriers which inhibit participation in studies of this nature. Once this was done, the participants were taken to the laboratory for the intervention. The major focus in the follow up discussion was to assess if there were changes in their views. An FGD guide containing well outlined questions was used during the discussions (Table 2). For each question, we asked about concerns relating to blood and biopsy specimens. We also explored any changes in views of the participants after going through the laboratory exposure intervention.

**Table 2:** Questions.

Sub topic	Questions
**Session One: Before the laboratory exposure intervention**	
Use of biological specimens	1. What do community members think biological specimens are used for after collection?What do you think biological specimens are used for after collection?
Storage of specimens	2. What are community members’ views relating to storage of specimens?What is your view relating to storage of specimens?
Willingness to donate specimens	3. What is your perspective regarding community members’ willingness to donate specimens?What would you say about your willingness to donate specimens?
Motivation to donate specimens	4. What issues motivate community members to donate specimens?What issues motivate you to donate specimens?What issues motivate community members to participate in studies of this nature?What issues motivate you to participate in studies of this nature?
Barriers to donating blood samples	5. What issues inhibit community members from donating specimens?What issues inhibit you from giving specimens?What issues inhibit community members from participating in studies of this nature?What issues inhibit you from participating in studies of this nature?
**Session Two: After the laboratory exposure intervention**	
Use of biological specimens	6. In which way has your view regarding what biological specimens are used for after collection changed?
Storage of specimens	7. How has your view relating to storage of specimens changed?
Willingness to donate specimens	8. What would say about your willingness to donate specimens?
Motivation to donate specimens	9. What is your comment regarding your motivation to donate specimens?
Barriers to donating blood samples	10. What is your comment regarding the barriers to donating specimens?What is your comment regarding the barriers to participating in studies of this nature?

#### Key informant interviews

Alongside these FGDs, three key informant interviews were conducted with staff (one female and 2 male) that are directly involved in recruiting study participants in the community. The staff are based at St Lawrence health facility. The main purpose of the interviews was to validate the themes that emerged in the FGDs by getting the views of the staff regarding their experiences in recruiting study participants.

#### The laboratory exposure intervention

Participants, including those who had previously donated specimens and those who had not but had volunteered to participate in the vaccine study, went through the intervention together. Non verbal as well as verbal expressions clearly showed that the participants had a keen interest in knowing more about the processes in the laboratory, and most communicated that they greatly appreciated being taken through the process. The intervention included demonstrations of the following processes.

1. *Processing blood:* One of the participants volunteered to have his blood drawn for demonstration. Blood was drawn into a plain tube Vacutainer (Becton Dickinson). The purposes of the different types of Vacutainers were also explained. The blood was allowed to clot at room temperature then centrifuged for serum separation. Using a Pasteur pipette the serum was aliquoted into storage tubes labelled with a code, date and sample type. It was explained that the codes were for confidentiality.
2. *Storing serum samples:* The serum samples were then stored in the −80°C freezer. The group was shown that samples were stored by study and by year. It was explained and demonstrated that some samples had been stored in the freezer for up to 15 years.
3. *Malaria screening:* The procedure for malaria diagnosis using thick and thin films was fully explained to the participants, and participants were able to see different sorts of blood cells. Venous blood was used from one of the participants who voluntarily donated the blood sample. Reagents and materials used were Giemsa stain, glass slides, applicator sticks, immersion and a binocular microscope.
4. *Storing biopsy specimen:* The group was then shown biopsies that had been taken from participants of a previous study. The samples had been stored in formalin from a study in 2008.

### Data analysis

All FGDs were recorded digitally and later transcribed verbatim by the first author. The first step in analysing data was the development of codes. The first author developed initial codes after reading the transcripts several times to develop a sense of the whole dataset. The codes were shared with the second author (MML, a biomedical scientist) for review. The authors separately reviewed codes by systematically comparing it to the dataset to arrive at the final code manual. Having agreed on the codes, the coding process, which involved matching the codes with segments of data selected as representative of the code, was carried out with NVIVO version 7 (QSR Australia). Codes were then grouped into categories – groups of content that share a commonality – and these were then developed into themes through a process in which all authors participated during the initial stages of developing the manuscript [17]. This involved interpreting the categories for their underlying meaning, and grouping categories according to patterns as reflected in Table 3.

**Table 3:** Codes, categories and themes.

Codes	Category	Themes
-Living healthy lives-Early detection of disease	Having a health body	Motivation for participation in invasive biomedical research
-Free medical screening-Free medical support	Accessing free medical services	
-Good research relationships-Duration of involvement in studies	Trust between the study participants andresearchers	
-Fears about of size of biopsy	What do they remove from my body?	Factors which might inhibit participation in invasive biomedical research
- Limited awareness on use biopsy- Involvement of non-African in the study	What do they do with my biopsy?	
-Effect of intestinal endoscopy-Misinterpretation of compensation	What happens to the body once the biopsyis removed?	
-Rumours about the specimens- Misunderstanding of free medical services-Concerns about quantity of blood	Blood used for satanic activities	
-HIV status and repeated health care consultation	Fear of being stigmatised as beingHIV positive	
-Women more willing to participate-Interference from husbands-Men think research is time-wasting	Gender inequality and perceptualdifferences	
-Better understanding of quantity blood required-Improved understanding of blood storage-Seeing old samples increasing confidence and trust	Enhanced understanding of useof specimens	Changes in perspective after the laboratory exposure intervention
- Satisfaction with the security of storage –andprocessing environment- Reduction in fears and insecurities-Commitment to sensitise the community	Increased willingness and confidenceto participate	

Data from the FGDs were then triangulated with other sources such as the information gathered through observations during laboratory processes and key informant interviews. Although the process is presented as a linear process, it is important to stress that this was an iterative process that involved continuous shifting back and forth from participants’ narratives to the researcher’s interpretation of what the informants meant [18]. The second, third, fourth and fifth authors participated in the triangulation process as they were responsible for conducting the observations. This process involved assessing the consistency and potential variations of findings by comparing data patterns across the material generated by different methods. The triangulation process showed that the major issues raised by participants such as concerns on the effect of endoscopy on the internal organs of the body as well as usage and storage of specimens were consistent across the different types of data. Finally, the themes or results were checked for validity during a meeting held in July 2013 at the University of Zambia for participants in another study which was attended by the key informants, members of the study team (including all authors) and other stakeholders who were not part of the study.

### Ethical issues

Ethical clearance to conduct this intervention was obtained from the University of Zambia Biomedical Research Ethics Committee as part of the original application for the vaccine study (UNZABREC 012-06-12). Written consent was obtained from all focus group participants or volunteers giving blood, including those who had provided consent in the earlier study. Verbal consent was also sought from the key informants and recorded using an audio recorder. Key informants did not provide written consent. The verbal consent procedure was approved by the ethics committee. Confidentiality, during and after the study period was guaranteed such that FGD and key informant responses, when reported to the PI and other study team members, were not attributable to any specific individual. Participation in the current study was voluntary. No financial incentives or other gifts were offered to participants except for transport refunds.

## Results

Social, economic, cultural as well as health related issues influence people’s attitudes towards participating in clinical trials that require invasive collection of biological specimens. In this section, these issues have been grouped into two main categories, namely motivating and inhibiting factors.

### Motivation for participation in invasive biomedical research

#### Having a healthy body

A dominant theme was that some people agreed to participate in biomedical studies because it helped them “live without falling sick”. All discussants reported that screening processes (always performed upon recruitment) allows for early detection of any diseases they might have, and enables them to adopt measures that will stop the disease from progressing. Screening also leads to medical advice about lifestyle and disease prevention, which study participants appeared to value. One participant stated it was through her involvement in a study that she learnt that she had a problem in the stomach which was then addressed. Another participant reported that he had been encouraged to continue participating in studies because it was through such involvement that he learnt that he had tuberculosis (TB).

*“If it was not for the study, I would have died. I had TB and I got very sick. People suspected that I was HIV positive because I was very slim. Thanks for the help from the researchers.”* (FGD 2, male participant 1).

#### Accessing free medical services

All the FGD participants who had participated in previous biomedical studies indicated that they were motivated to do so because participation guaranteed them free access to a range of health services whenever they were sick. Furthermore, they stated that once screened and found with a health problem, they would have free medical support. The FGD participants who had never previously participated in biomedical research also reported that they had been inspired to seriously consider participating in trials because of the desire for free medical services, one of the benefits of such participation.

*“I have participated in giving blood and biopsy specimen because we are given free treatment once we get sick. I think this is what some members of the community admire.”* (FGD 1, female participant 3).

Key informant interviews also confirmed that participants willingly participate in such studies because they hope to access free medical services. Key informants stated that the majority of those who participated in biomedical studies inquired about the possibilities of accessing free medical services during the consent process.

#### Trust between the study participants and researchers

Trust between the researchers and study participants seemed to be another motivating factor cited by the study participants. This was raised by those who had participated in previous studies. They reported that they had undergone more than one intestinal endoscopy partly because of the good relationship which they had developed with the Principal Investigator. They reported that the research team had been operating a clinic in their community/compound for more than thirteen years, a situation which had made them trust their activities.

*“We have worked with the doctor (Principal Investigator) for more than five years. We trust him and this is why we give him our blood whenever he asks for it.”* (FGD 1, female participant 1).

#### Inhibiting factors which might constrain participation in invasive biomedical research What do they remove from my body?

The FGD participants who had been involved in previous studies did not express any concerns over giving of blood samples for research purposes as they could actually see the blood being drawn out from their bodies. However this was not the case with regard to endoscopic procedures as the study participants are given medication (sedation with benzodiazepines) which means that they have no recall of what happened. This lack of awareness has resulted in speculation about what it is that is taken from the body, and more so the size of the biopsy taken. This has led to some community members, even those who have never had an endoscopy, insinuating that big pieces of flesh are removed from the body leaving big sores where they have been taken from.

*“So my question has been: when they put the tube inside me, what is it that they remove from inside my body-this is my biggest anxiety?”* (FGD 4, male participant 2).

#### What do they do with my biopsy?

A dominant concern related to the use to which the biopsy sample *(‘kanyama’ in the local language, translated into ‘flesh’ in English*) would be put.

*“I have heard that when they put the tube inside the body, they remove a piece of flesh (‘kanyama’), so my question is: what do they do with the flesh?”* (FGD 4, male participant 4).

In FGDs as well as key informant interviews, we delved into some community beliefs about what they thought these samples were being used for. One participant reported that it was rumoured the samples are taken out of the country and used for non-medical purposes such as fishing. Further discussions showed that several FGD participants had come across someone who believed in this view. The involvement of non- Africans in the study, such as the Principal Investigator, was felt to contribute to this concern. This is a belief of those when they do not know the PI or key team members. While several of them admitted having concerns regarding the use of the biopsies, most of the participants did not explicitly state whether or not they believed in this view.

*“Lets be honest. In the compound, people say that they use the flesh to catch shark in the ocean. They say that the shark has a precious stone which attracts a lot of money once it is sold.”* (FGD 1, female participant 2).

#### What happens to the body once the biopsy is removed?

Another concern raised, especially from those who had undergone an intestinal endoscopy, was the effect the endoscopy has on the internal organs of the body. They were under the impression that the procedure would result in a wound or sore in the intestine during or after the procedure. This came about as a result of a discussion during one of the recruitment meetings where they were informed that in the event that something did go wrong during the endoscopic procedure they would be compensated for any health complications that may result from it. Some respondents interpreted this compensation as for the wound which develops from the site of the biopsy. However, they were quick to mention that at the same meeting, they were told that previous studies had shown that the risk of developing health complications was quite low.

*“The other concern is that, when you remove the flesh, what remains there? Doesn’t this result in a wound? Then, if so, does the same wound heal?”* (FGD 3, male participant 1).

#### Blood used for satanic activities

Insecurities about drawing of blood samples for use in studies resulted from the belief that blood is used in the practice of Satanism and as such they feared that their blood could be used for the same purposes. It was reported that some members in the community believed blood donors would end up experiencing misfortune in their families such as sickness or death of their family member, as the process of giving blood was perceived as an act of sacrificing the lives of family members or of self. Although they acknowledged that not everyone shared this view in the community, several of the participants had been warned by community members not to donate blood samples for the study because of Satanism.

*“Let me give an example, I had a girl who was in grade 12 and had a problem with her leg…., so the doctor (Principal Investigator ) advised me that she be put on treatment. We did that but unfortunately my child died.” “Do you know what people said?” “They told me that my child died because I was participating in the research activities, which are satanic.”* (FGD 1, female participant 1).

It was further reported that some of the community members substantiate this belief by suggesting that the free medical services which some study participants access once they join the study are actually payments given in exchange for the donated blood. Some members of the community stated that researchers transport blood outside the country for sale. While some FGD participants said that they did not believe in such stories, others informed the group that they were once part of groups which spread rumours that all those who were part of such studies were Satanists. One participant even mentioned the name of some of the individuals that she had warned not to join studies as she believed that they were practicing Satanism.

*“I used to say those people who give blood are Satanists. I recall warning this one (pointing at another FGD participant) not to be part of the study. I told her that it is an activity for Satanists.”* (FGD 2, female participant 3).

Detailed analysis of responses suggests that these suspicions often increase when people are required to give more than one Vacutainer of blood. It was reported that they often wonder what happens with the other tubes, as one small bottle should be sufficient for doing tests. One FGD member stated that she had been banned from participating in the women’s group at church because the others believed that she practised Satanism by donating blood for research activities.

#### Fear of being stigmatised as being HIV positive

In key informant interviews, it was reported that potential participants were afraid of being labelled as HIV positive. HIV is a common reason for repeated health care consultation, so fear of stigma may lead to avoidance of frequent attendance at health care facilities.

#### Gender inequality

Although the complexity of issues surrounding undergoing an intestinal endoscopy as well as drawing of blood apply to both sexes, all FGD participants and key informants agreed that women were more willing to participate in biomedical research than men. One possible reason given was that it is mainly women who care for sick people and so value activities aimed at improving health. Two FGD participants reported that their husbands stopped them from undergoing an intestinal endoscopy while one reported that her husband stopped her from participating in a clinical trial until she developed a health problem. The men explained that some men think that participation in research is an unproductive way of using time as men are supposed to spend time raising funds for the family.

### Changes in perspective after the laboratory exposure intervention

#### Enhanced understanding of use of specimens

Having been shown what happens to blood samples once they reach the laboratories, all participants indicated that they now understood why at times they are required to donate more than one bottle of blood or why blood is stored for some time in the laboratory. Furthermore, fears about where and how the samples are stored were quickly overcome. Participants stated that seeing old samples being properly refrigerated was proof enough that samples were not being exported for sale. As participants were being shown the stored samples, especially biopsies, one could easily observe that they were surprised that it was possible to keep samples for such a long time. Some participants seemed relieved that the biopsy specimen was smaller (actually about 3×1 mm) than was being projected by some members of the community. One of the participants openly showed her excitement upon seeing the biopsy specimen which was taken from her close to five years before. The participant recognised that the code on the biopsy was hers.

*“Yes, this is my code… I can’t believe what am seeing!… this was taken a long time ago from my stomach… so what people have been telling us that these people misuse samples is not true.…rumours can mislead someone.”* (FGD 1, female participant 1).

#### Increased willingness and confidence to participate

When FGD participants saw how the samples were being processed and stored in a secure environment they stated that they would consent more freely to future biomedical research. Those who had never previously been involved in such studies (but had already given consent to the vaccine study and were waiting for their turn in endoscopy) indicated that they were now more than before ready to undergo an intestinal endoscopy. This attitude was a sharp contrast to their attitude before being taken to the laboratories, which was characterised by doubts and hesitations. For those who had been involved in such practices before, they stated that the process further strengthened their desire to continue being part of such activities. Most importantly, they acknowledged that this had erased most of their fears and insecurities about blood and biopsy specimen. Most participants went as far as to suggest that they would all commit themselves to go and dispel the rumours and misconceptions about the use of blood and biopsy samples.

*“I am now ready to give my biopsy (kanyama) for the study…, it is actually a tiny thing… nothing much to worry about. I do not just want to be giving those urine samples.”* (FGD 1, female participant 3).

## Discussion

The paper has discussed the dynamics that shape people’s attitudes towards giving of blood samples in Misisi compound of Lusaka. Cultural interpretations, religious values, social stigmatisation and health concerns are some of the main issues which shape people’s attitudes towards consenting to participate in invasive biomedical research and clinical trials. We have obtained clear evidence that immersion of participants in a laboratory for a full day, followed by group discussions, can enhance understanding of certain biomedical procedures. Notwithstanding that most of our participants have little or no scientific education and a few cannot read in any language, the demonstration of key processes and samples had a strong demystifying effect. The reassurance provided was palpable, and it is our belief that the enhanced understanding of laboratory practices can only enhance the validity of consent, hinging as it does on comprehension of what is really involved.

Culture, which is often learnt through the process of socialisation, plays a great role in defining and moulding people’s behaviour and attitude. “Within the environment into which the child is born, he learns the language, customs attitude and the various ways in which things are done in his society by observing his surroundings” [19]. These beliefs certainly include perceptions relating to blood. Tissue biopsies are a modern phenomenon, but nevertheless when there is an information gap, rumours will develop to fill that gap.

There are many rumours about blood in Africa, which have been shaped by different histories and political, social and economic structures within countries [20]. Blood is often viewed as sacred. It is only in living memory that it has become accepted that it can be drawn out of the body under special circumstances for example during medical examination when a person is sick [3]. It is for this reason that respondents in this study expressed fear of donating blood and tended to associate the practice with Satanism. This finding resonates with other reports of fear that researchers use blood samples for rituals, and blood collection is viewed with suspicion in some African communities. Such rituals would have the potential of creating misfortunes in families of those involved, and this understandably generates fear [3].

Beliefs about certain practices define social relations and influence how people react towards certain activities in the community. It is important to note that acting outside culturally defined behavioural norms usually results in being labelled a ‘deviant’ [19]. Such a label would attract social discrimination and subsequently loss of social support. This could explain the experience of the woman who was reportedly excluded from church activities for participating in a biomedical research study. Such exclusion from family, community or religious activities would create severe insecurity in individuals in most African countries where socio-cultural networks are strong, and “the individualism as a way of being has little or no place in societies which have strong sense of kin and community ties, where individuals meet their needs on the basis of shared morality of claims and obligations” [21]. Several people define their identity and social support “by participating in the identity of a collectivity. This identification is often expressed in exalted, mystical terms. The real me is joined to the spiritual life of a community” [22]. For, Zambia, one of the key collective issues is religion, as Zambia is predominantly Christian [23]. Some religions such as Jehovah's Witnesses believe that the Bible prohibits blood donation and transfusion [24]. Fear of exclusion encourages conformity to cultural norms and values [19], a situation which, if not fully understood and addressed, has the potential of negatively affecting giving of informed consent for clinical trials.

Fear of negative effect on health is a major constraint on willingness to participate. In one FGD it was reported that a woman’s daughter’s death was attributed to her involvement in a research study. Helman [25] notes that in non-Western societies, illness maybe due to the active intervention of an agent, such as a supernatural being (a god) or human being (witch or sorcerer). For instances, in a number of societies, the out-break of a disease with no cure or origin may be attributed to the committing of an offence against one’s spirits, the ancestors or the gods, or an omission of duty on the part of the infected person [26], [27]. For example, at different times in Ghana, the outbreak of diseases such as tuberculosis, measles and guinea worm has been attributed to supernatural causes [28]. It has been stated that “the invocation of ‘witchcraft’ provides ways of answering the questions: why me? or her/him?” [29]. Self-perception of being unwell, which in the context of this study include the fear that the biopsy may create a wound in the body [30], may also negatively affect giving of informed consent in studies.

Apart from communities being anxious that blood would be used for ritual activities, our findings suggest that involvement of non Africans in studies can exacerbate concerns that biological specimens could be transported out the country and used in business transactions. These concerns are not unique to this study as other workers have found that resistance to participation in clinical trials was more pronounced if the community perceived the African researchers as associated with ‘white’ researchers [31].

Stigmatisation by association with HIV may be another constraint. Respondents stated that the processes for testing of blood in biomedical studies was compared to HIV testing processes by several community members. Regular attendance at health facilities was likened to regular check ups that people who are living with HIV go through. Banteyerga et al., note that HIV/AIDS stigma exists and that it has an effect on the choices that people make including health seeking decisions. HIV/AIDS stigmatisation, according to Banteyerga et al. [32] takes different forms, which include “verbal,” for example laughter or ridicule; “social exclusion,” such as loss of social security or belonging; and “loss of identity,” which encompasses shame or existential and cultural insecurities. It is a mark of underperformance by local health services that HIV is perceived as the only legitimate reason for regular health care, and long term care for non-communicable disease, which should be the norm in the health sector, is regarded as exceptional. While much progress has been made in reducing the HIV stigma, clearly much more needs to be done. This sort of constraint could probably not be expected to respond to a laboratory exposure intervention, and indeed this is what we found.

Gender inequalities may also affect the giving of informed consent. One of the cultural issues surrounding masculinity and femininity include the need for permission for women from men, more so in the case of couples, before participating in clinical trials. Some women, especially those who are married, had difficulty getting support from their husbands who would not allow them to participate. Several studies in Africa have shown that a real man is supposed to exercise authority over women or risks being perceived as not ‘man enough’ [33]. As with HIV, this constraint did not respond to our intervention.

Other factors which have been documented, though not captured in our discussions include fear of hospitals, unpleasant experiences following blood draws, fear of needles, and pain [30]. A study in India showed that the most common reason for not donating blood was the perception of a harmful effect of donation on the body. Cultural beliefs were however not commonly cited as factors although the respondents mentioned that collecting blood from children and pregnant women could lead to serious health consequences. Several respondents questioned why blood would be taken from participants who were not sick [9]. On the other hand, this might explain why the spacious and clean dedicated facilities in which they are seen for the purposes of the study might encourage people to participate.

Overall, the participants in this study, like other studies [30], showed a lack of awareness of the processes of drawing blood and taking biopsy specimens, storage of samples, and usage of the specimen as causes of fears and misconceptions about participating in studies that require invasive procedures. The intervention we implemented had a dramatic effect on these anxieties. This resonates with Boahen et al. [3] ’s view that it is important to complement oral explanations with a visual presentation explaining what the blood-draw procedure entails and the uses if people’s ability to give informed consent are to be improved. We have not ascertained the extent to which the effect of our laboratory exposure intervention could be replicated by a video demonstration, but our impression was that the immediacy of the immersion we used might have an impact beyond the purely visual.

## Strengths and Limitations of the Study

The strength of the study was enhanced through the use of multiple methods to collect data. Triangulating data collection methods helped in developing an account that is rich and comprehensive [34]. Credibility of the findings was enhanced through thoroughly documenting the research process, transcribing and reviewing all interviews. In addition sharing the results with the key informants, members of the study team and others stakeholders who were not part of the study during a meeting related to another study held in July 2013 at the University of Zambia helped in clarifying the findings [34] and also provided additional data [35]. Our findings cannot be generalized to high income and more educated groups, as the participants in our study were all drawn from the disadvantaged community living in Misisi. The other limitation is that the study sample size was small and it is also possible that the sample of participants included in the study are those most likely to be interested in participating in this sort of research. Despite these limitations, the inclusion of key informants who were responsible for recruiting participants for the original study as well as questions regarding FGD participants’ perspectives of factors that facilitate or inhibit other community members from giving consent provided some insights which could apply to some of the people who may not have been interested in participating in the study. In general, the rich description of phenomena [36], factors that motivate and hinder people from giving blood and biopsy specimen, contributes to the knowledge base on improving informed consent and may provide a basis for analytic generalizations that could provide useful insights in similar settings.

## Conclusion

We have found that peoples’ ability to provide informed consent in studies that require the use of blood and biopsy specimen is shaped by various contextual factors, some of which may beyond an individual’s capacity. These include the desire to access free health services, being aware about ones’ health status and adopting healthier lifestyles based on the advice from doctors. However, limited knowledge regarding what happens during the processes of giving blood and biopsy specimen, cultural beliefs/concerns about the use of the samples (.e.g. rumours that blood is used for satanic activities and biopsies for fishing sharks), gender inequality as well as fear of social stigmatisation and loss of social support were some of the factors which may inhibit full participation.

We observed that taking participants though the various laboratory processes significantly changes peoples’ attitudes towards giving blood and biopsy specimen by increasing awareness levels about not only the laboratory processes but also the benefits associated with giving specimen. We propose that selecting a group of members from target community and engaging them in a laboratory exposure intervention can be a useful addition to consent processes for biomedical research in communities with low scientific exposure. This could be followed by appropriate motivational campaigns based on the input from the study participants aimed at addressing concerns within the community. This is a potential tool for enhancing consent for biomedical research in developing countries which will be needed for developing approaches to emerging health problems, new and old. We therefore recommend an additional longitudinal analysis to see the effects of the intervention for other future participants.
